# Non-classical Vitamin D Actions for Renal Protection

**DOI:** 10.3389/fmed.2021.790513

**Published:** 2021-12-07

**Authors:** Adriana S. Dusso, Kevin T. Bauerle, Carlos Bernal-Mizrachi

**Affiliations:** ^1^Division of Endocrinology, Metabolism and Lipid Research, Department of Medicine, Washington University School of Medicine, St. Louis, MO, United States; ^2^Department of Medicine, VA Medical Center, St. Louis, MO, United States; ^3^Department of Cell Biology and Physiology, Washington University School of Medicine, St. Louis, MO, United States

**Keywords:** klotho, ADAM17, FGF23, hypertension, ACE2, systemic inflammation, chronic kidney disease

## Abstract

Chronic Kidney Disease (CKD), a disorder that affects 11% of the world's population, is characterized by an acceleration in skeletal, immune, renal, and cardiovascular aging that increases the risk of cardiovascular mortality by 10- to 20-fold, compared to that in individuals with normal renal function. For more than two decades, the progressive impairment in renal capacity to maintain normal circulating levels of the hormonal form of vitamin D (1,25-dihydroxyvitamin D or calcitriol) was considered the main contributor to the reduced survival of CKD patients. Accordingly, calcitriol administration was the treatment of choice to attenuate the progression of secondary hyperparathyroidism (SHPT) and its adverse impact on bone health and vascular calcification. The development of calcitriol analogs, designed to mitigate the resistance to calcitriol suppression of PTH associated with CKD progression, demonstrated survival benefits unrelated to the control of SHPT or skeletal health. The exhaustive search for the pathophysiology behind survival benefits associated with active vitamin D analogs has identified novel anti-inflammatory, anti-hypertensive, anti-aging actions of the vitamin D endocrine system. A major paradigm shift regarding the use of calcitriol or active vitamin D analogs to improve survival in CKD patients emerged upon demonstration of a high prevalence of vitamin D (not calcitriol) deficiency at all stages of CKD and, more significantly, that maintaining serum levels of the calcitriol precursor, 25(OH)vitamin D, above 23 ng/ml delayed CKD progression. The cause of vitamin D deficiency in CKD, however, is unclear since vitamin D bioactivation to 25(OH)D occurs mostly at the liver. Importantly, neither calcitriol nor its analogs can correct vitamin D deficiency. The goals of this chapter are to present our current understanding of the pathogenesis of vitamin D deficiency in CKD and of the causal link between defective vitamin D bioactivation to calcitriol and the onset of molecular pathways that promote CKD progression independently of the degree of SHPT. An understanding of these mechanisms will highlight the need for identification of novel sensitive biomarkers to assess the efficacy of interventions with vitamin D and/or calcitriol(analogs) to ameliorate CKD progression in a PTH-independent manner.

## Introduction

The vitamin D endocrine system is critical for human health, and a structurally normal kidney is essential to maintain the functional integrity of the vitamin D endocrine system. In the general population, vitamin D deficiency is associated with an increased relative risk for cardiovascular and all-cause mortality (1, 2). Accordingly, in chronic kidney disease (CKD), a disorder that affects 11% of the world's population, there are progressive reductions in renal capacity to produce the active form of vitamin D, 1,25-dihydroxyvitamin D (calcitriol), and a surprising inability to maintain circulating vitamin D levels, resulting in an accelerated immune, skeletal, renal and cardiovascular aging. The end-result is a 10–20-fold increase in cardiovascular morbidity and mortality, compared to those in gender- and age-matched individuals with normal renal function (3, 4).

Defects in mineral metabolism, including intestinal calcium absorption and renal phosphate excretion, are best characterized features of CKD associated with vitamin D/calcitriol deficiency. These defects in mineral metabolism contribute to the onset and progression of secondary hyperparathyroidism (SHPT) and its associated abnormal bone remodeling including defective mineralization, increases in bone resorption and fracture risk and the propensity to vascular and soft tissue calcifications (5). It is also important to note that hyperphosphatemia is negatively correlated with lifespan in mammals. Maintenance of phosphate homeostasis requires a complex endocrine network involving PTH, vitamin D, FGF 23, and Klotho to provide appropriate signals to the kidney, parathyroid glands, gut, and bone. While hyperphosphatemia is known to stimulate pro-inflammatory, pro-aging and pro-fibrotic signals to exacerbate renal and cardiovascular damage (6), the mechanisms by which active vitamin D induces expression of the *FGF23* and α*-klotho* genes to attenuate the pro-aging effects of hyperphosphatemia and maintain the plethora of anti-aging/pro-survival actions of renal and circulating klotho are not fully understood (7).

The prevalence of vitamin D deficiency increases with advancing CKD (8) and has a more severe impact on survival than that of calcitriol deficiency (9). This finding has challenged 30 years of clinical experience, where calcitriol was exclusively administered to control SHPT. In fact, particularly pertinent to topic of this review on vitamin D prevention of CKD progression, levels of the calcitriol precursor 25(OH)D above 23 ng/ml and not of calcitriol are independently associated with reno-protective actions (10). Since neither calcitriol nor its analogs correct vitamin D deficiency, and in view of the controversies from prospective trials regarding the actual efficacy of vitamin D supplementation strategies to improve survival in individuals with normal renal function, or SHPT and kidney injury in early CKD, the overall goal of this chapter is to update our understanding on (1) CKD-induced defects in systemic and local vitamin D bioactivation and calcitriol actions and (2) outline approaches to improve strategies to effectively ameliorate CKD progression independently of the degree of SHPT by attenuating its strongest inducers, systemic inflammation, hypertension, renal and cardiovascular damage. Special focus will be directed at the pathophysiology driving calcitriol/VDR-mediated reduction in pro-inflammatory and hypertensive signals unrelated to klotho reductions that promote multi-organ damage, including:

1. Hypertension-driven renal and vascular damage unrelated to the suppression of renin gene expression
2. Immune cell-driven systemic inflammation and oxidative stress-mediated multi-organ damage
3. Inflammation-induced renin-driven hypertension.

This mechanistic knowledge is a mandatory first step to evaluate the accuracy of current biomarkers of the severity of CKD progression and of the response to vitamin D therapy.

## Defective Vitamin D Bioactivation to Calcitriol in CKD

Vitamin D is not truly a vitamin because, in mammals, the sun light [ultraviolet B range (UV-B)] converts the skin precursor 7-dehydrocholesterol into vitamin D_3_ (cholecalciferol) (11). This conversion is completely prevented by sunscreen (12). The next step occurs primarily in the liver, where two cytochromes p450 enzymes, mitochondrial CYP27A1 and microsomal CYP2R1, hydroxylate cholecalciferol at the 25-position to produce 25-hydroxyvitamin D (25(OH)D), the main circulating vitamin D metabolite, with a biological half- life of 15–18 days. Since only mutations in CYP2R1 result in severe vitamin D deficiency (13) (serum 25(OH)D levels below 10 ng/ml), CYP2R1 is considered the most critical vitamin D-25-hydroxylase. Even though skin and parathyroid cells, immune cells and endometrial cells express 25-hydroxylases (14, 15), neither their distribution in other tissues nor the regulation of their expression or activity are fully understood. Nevertheless, because vitamin D measurements require assays too complex for routine biochemistry laboratories, and 25-hydroxylases efficaciously convert most circulating vitamin D into 25(OH)D (16), measurements of 25(OH)D levels are used to estimate the vitamin D status of an individual.

An important clinical consideration is that only mass spectrometry accurately measures 25(OH)D levels. Most common assays used worldwide have a 100% cross reactivity of 25(OH)D with 24,25(OH)_2_D, a metabolite that marks the first step in 25(OH)D degradation, and consequently, there is an overestimation of vitamin D status.

**Figure 1:**
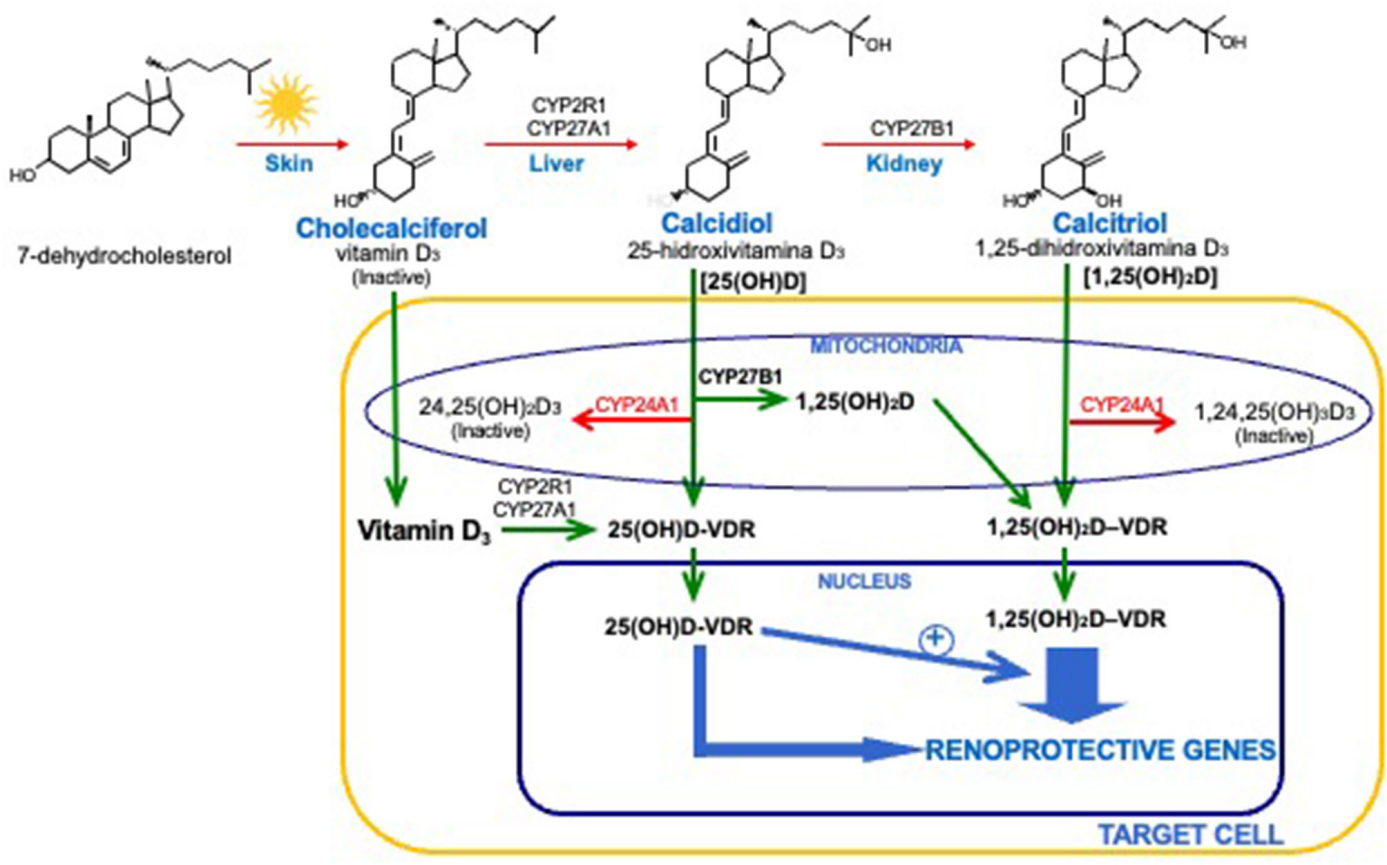
Vitamin D Bioactivation and calcitriol/VDR renoprotective actions. Systemic and intracellular vitamin D activation to 25(OH)D and calcitriol; intracellular balance between activating CYP2R1/CYP27A1 or CYP27B1 with catabolizing CYP24A1 responsible for final 25(OH)D and calcitriol concentrations for VDR binding and activation, and 25(OH)/calcitriol-synergy to enhance calcitriol-VDR transcriptional regulation of renoprotective genes.

The most critical and tightly regulated step in vitamin D bioactivation occurs mainly, although not exclusively, in renal proximal tubules, where the mitochondrial cytochrome p450 CYP27B1 catalyzes the 1α-hydroxylation of 25(OH)D to produce calcitriol, the most active endogenous vitamin D metabolite (17). Under physiological conditions, this renal conversion is the main, if not the only, contributor to circulating calcitriol. Thus, vitamin D is actually an inactive pro-hormone whose biological actions require a two-step bioactivation to 1,25-dihydroxyvitaminD (1,25D or calcitriol), a potent steroid hormone (Depicted in Figure 1) (11).

In contrast to the 25-hydroxylases, where the enzymatic activity is not closely regulated, CYP27B1 is tightly regulated to maintain serum calcitriol within the narrow limits required to avoid hypercalcemic and hyper-phosphatemic episodes. CYP27B1 is induced by PTH and suppressed by FGF23 and klotho. Calcitriol itself also controls its own circulating levels through a feed-back suppression of its own synthesis, but mostly through the induction of CYP24A1 gene expression. This enzyme, responsible for calcitriol and 25(OH)D degradation, is constitutively expressed in the kidney and strongly induced by either calcitriol or its analogs in every vitamin D target tissue, thus reducing the toxicity associated with high circulating calcitriol (18). Accordingly, to maintain normal circulating calcitriol levels, PTH and FGF23 suppress and induce CYP24A1, respectively.

Numerous other cell types express CYP27B1 and produce calcitriol in a cell/tissue specific manner. This extrarenal calcitriol production is of high clinical relevance beyond CKD. In fact, the multiple health disorders associated to vitamin D deficiency in the general population occur despite normal serum calcitriol. The low circulating 25(OH)D levels during vitamin D deficiency (below 20 ng/ml) reduce local calcitriol synthesis by non-renal cyp27B1s, thus compromising the plethora of tissue-specific pro-survival actions of calcitriol.

CKD severely compromises renal and extrarenal vitamin D bioactivation to calcitriol starting with an impaired photo-activation of the skin precursor 7-dehydrocholesterol to cholecalciferol (19).

The high incidence of vitamin D deficiency at all stages of CKD (8) emphasizes the critical role of normal kidney function in maintaining normal serum 25(OH)D by facilitating the reabsorption of 25(OH)D that has been previously filtered at the glomerulus. 25(OH)D is a lipid soluble molecule that circulates bound to its main carrier, the vitamin D binding protein (DBP), a low molecular weight protein (60 KDaltons), similar in mass to albumin. The 25(OH)D/DBP complex is filtered at the glomerulus and its reabsorption occurs at proximal tubules and requires adequate tubular cell levels of the endocytosis receptor, megalin (20). In CKD, an early loss of renal megalin (21) impairs the renal uptake of 25(OH)D compromising non only mitochondrial calcitriol production, but also 25(OH)D recycling back to the circulation to ensure adequate extrarenal calcitriol synthesis. Interestingly, vitamin D induces the expression of renal megalin (22). Thus, the early correction of vitamin D deficiency in CKD recommended by KDIGO guidelines may attenuate renal megalin reductions, thereby improving renal and extrarenal calcitriol production.

The actual contribution of impaired uptake of 25(OH)D on renal calcitriol production was demonstrated by a strong correlation between serum calcitriol and 25(OH)D levels in advanced CKD patients with a glomerular filtration rate below 25 ml/min (23, 24), which does not exist in individuals with normal renal function. Importantly, in addition to defective substrate availability for calcitriol production in CKD, a higher fragmentation of PTH in the parathyroid gland, elevations in FGF23, and the accumulation of uremic toxins further reduce CYP27B1 expression to exacerbate calcitriol deficiency, as reviewed in (17).

Importantly, 25(OH)D supplementation to hemodialysis patients can normalize serum calcitriol, even though renal mRNA levels for CYP27B1 should be markedly reduced by high serum FGF23 (24). The contribution of extrarenal calcitriol production cannot be fully disregarded as FGF23 increases rather than decreases parathyroid CYP27B1 expression (25). Similarly, despite the increases in CYP24A1 mRNA induced by high FGF23, serum levels of 24,25-dihydroxyvitamin D in non-supplemented or in cholecalciferol supplemented patients were persistently lower than normal (26, 27). Clearly, in advanced CKD, the activity of either enzyme fails to reflect FGF23 control of the respective genes—that is, the damaged kidney fails to respond to regulation of renal calcitriol production by FGF23.

Furthermore, as will be discussed in the section of vitamin D control of systemic inflammation, CKD also impairs the substrate availability for immune cell calcitriol production.

## Defective Calcitriol-Vitamin D Receptor (VDR) Actions in CKD

Once calcitriol is synthesized in the kidney or extrarenally, most of its biological actions are mediated by binding to the VDR. VDR binding of calcitriol promotes a conformational change in VDR that facilitates heterodimerization with the retinoid X receptor (RXR) and the binding of the VDR/RXR complex to vitamin D responsive sequences (VDREs) in the promoter regions of vitamin D responsive genes to regulate gene expression (18). There are multiple simultaneous rather than a single site for binding of the ligand-activated VDR/RXR complex up or downstream from the transcription start site of a target gene (28) which are juxtaposed by chromatin looping, thus facilitating the recruitment of basic transcription factors, co-activator and/or co-repressor molecules to multiply the potency of VDR-RXR/VDRE complexes at regulating vitamin D responsive genes (29).

These calcitriol/VDR-DNA interactions activate/repress the expression of the 500–1,000 genes responsible for the survival benefits associated with normal vitamin D status. The most well-characterized calcitriol/VDR actions in nephrology relate to the control of the calcium/PTH and phosphate/FGF23-klotho axes, including the suppression of PTH synthesis and the induction of the phosphaturic hormone FGF23, the longevity gene klotho, and the calcium channel TRPV6 in enterocytes, the rate limiting step in intestinal calcium absorption. Other upregulated genes include the parathyroid calcium sensing receptor, which controls the responsiveness of the parathyroid gland to serum calcium and senses high circulating phosphate suppressing its inhibitory effect on PTH (30), and the receptor of the canonical Wnt pathway LRP5 in bone (18).

Some of these calcitriol/VDR target genes code for microRNAs (31), short (18–25 nucleotides) non-coding RNAs that control the expression of more than 30% of the genes in the genome by binding the 3' untranslated region of target mRNA, decreasing either their stability or protein translation. Calcitriol/VDR induction of miR-145 is of particular interest. miR-145 is the most abundant microRNA in normal vascular smooth muscle cells and may contribute to vascular protection, as miR145 is downregulated in proliferative vascular diseases (32) and also in uremia and hyperphosphatemia (33, 34). Reductions in miR145 directly augment ADAM17 gene expression, an enzyme which is critical for (1) the severity of parathyroid hyperplasia in the course of CKD (35), (2) release of TNFα that enhances systemic inflammation and multiorgan damage (36), and (3) angiotensin II-driven renal damage at early CKD stages (37).

The calcitriol/VDR complex also indirectly controls certain “apparent” target genes through the regulation of the expression of an essential inducer or repressor gene. Important examples related to attenuation of CKD progression include induction of C/EBPβ to facilitate suppression of parathyroid ADAM17 gene expression, which is critical to attenuate hyperplastic growth of parathyroid tissue (38). Additionally, VDR activation results in a 30-fold induction of FGF23 expression, critical for the renal handling of excess phosphate, which is markedly attenuated by inhibiting new protein synthesis. This finding suggests that the increase in FGF23 expression by calcitriol/VDR occurs via a yet unknown mediator (39).

Undoubtedly, the intracellular levels of both calcitriol and VDR determine the magnitude of calcitriol/VDR complex formation and the efficacy for direct or indirect control of target genes expression by the calcitriol/VDR complex, and both are reduced in CKD (17). Decreases in cellular levels of the VDR partner for the regulation of gene expression, the retinoid X receptor (RXR), as well as increases in systemic levels of uremic toxins reduce calcitriol/VDR-RXR binding to DNA, further impairing the response of CKD patients to vitamin D therapy [Reviewed in (40)].

Therapeutically, the development of calcitriol analogs that selectively maintain the benefits of VDR activation with less calcemic or phosphatemic activity (41) has helped improve outcomes, by allowing a safer escalation of analog dosage to counteract the progressive resistance to therapy caused by CKD-induced VDR reductions (42). Two of these “so called” analogs, 1-α-hydroxyvitamin D3 and 1-α-hydroxyvitamin D2 (doxercalciferol), are calcitriol precursors, as they can be activated to 1,25-dihydroxyvitamin D3 or D2, respectively, in the liver, through their hydroxylation at carbon 25. On the other hand, paricalcitol and maxacalcitol are true calcitriol analogs, structurally different compounds that maintain calcitriol selective actions in the control of SHPT with less adverse effects on calcium and phosphate metabolism as reviewed in (43). There are important clinical considerations for interventions using high doses of calcitriol, calcitriol precursors or its analogs: (1) They are incapable of correcting underlying vitamin D deficiency, and (2) their induction of CYP24A1 expression may further reduce not only systemic levels of 25(OH)D for local calcitriol production, but also intracellular calcitriol for VDR activation (see Figure 1).

A previously unrecognized synergy between 25(OH)D and calcitriol for VDR activation could be exploited to safely improve clinical outcomes in CKD without escalating calcitriol (analog) doses (see Figure 1). Studies in the CYP27B1 null mouse (44), which lack the capacity to convert 25(OH)D to calcitriol, and using 25(OH)D analogs chemically modified to prevent hydroxylation at carbon 1 (45, 46) have demonstrated that 25(OH)D can activate the VDR directly, and more importantly, it synergizes with calcitriol activation of the VDR. This 25(OH)D/calcitriol synergy, achieved by normalizing serum 25(OH)D levels, was shown sufficient to overcome the parathyroid resistance to low doses of the calcitriol analog paricalcitol caused by VDR reductions and accumulation of uremic toxins, even in hyper-phosphatemic experimental CKD (38). Thus, in tissues like the parathyroid glands and monocyte macrophages expressing 25-hydroxylases, and whose activities are not as tightly regulated as renal CYP27B1 and CYP24A1, appropriate vitamin D supplementation will help promote synergistic 25(OH)D/calcitriol interactions by increasing intracellular 25(OH)D levels with minimal, if any, impact on systemic calcium homeostasis. If the capacity for extrahepatic conversion of circulating cholecalciferol to 25(OH)D is tissue specific, current recommendations to achieve certain 25(OH)D levels to improve outcomes may not be accurate. A recent comprehensive review updates our understanding of the epidemiology of native hypovitaminosis D in CKD, current available therapeutic interventions and the existing challenges to achieve an appropriate correction of vitamin D deficiency/insufficiency, not only to ameliorate the progression of SHPT, but also to confer renal and cardiovascular protection improving outcomes (47). A better understanding of the modulators of the tissue specific expression and activity of 25-hydroxylases in CKD could improve current recommendations to enhance the survival benefits of a normal vitamin D status in these patients.

## Lessons from the Defective Calcitriol Suppression of PTH Synthesis and Parathyroid Hyperplasia in CKD

The parathyroid gland is the calcium sensor of the body and one of the best studied targets of vitamin D actions, where it inhibits PTH synthesis and secretion and suppresses hyperplastic parathyroid cell growth, thus attenuating bone loss and the propensity to develop vascular calcification that increases mortality rates in the course of CKD. Indeed, in hemodialysis patients, the COSMOS study, a large European prospective study with a 3 year follow up on the clinical handling of more than 6,000 CKD-5D patients, has provided an optimal range of serum PTH that associates with the lowest relative risk of mortality (48). Furthermore, COSMOS has also corroborated the survival benefits associated with correction of serum PTH to achieve values within the optimal range.

Hypocalcemia, hyperphosphatemia and vitamin D deficiency are the main causes of SHPT (49). The calcitriol/VDR complex suppresses PTH synthesis through a direct negative regulation of the PTH gene promoter (18). Vitamin D deficiency (50) and progressive reductions in serum calcitriol in the course of CKD (51) also impair the response of the parathyroid gland to calcium due to reductions in parathyroid content of the calcium sensing receptor (CaSR), a gene directly stimulated by the calcitriol/VDR complex (52). In addition to the control of the calcium/PTH axis, the calcitriol/VDR complex induces FGF23 synthesis by cells of the osteoblastic/osteocyte lineage, providing an additional mechanism to reduce PTH secretion if there is sufficient parathyroid klotho (53), another gene induced by the calcitriol/VDR complex (18).

In individuals with normal renal function, the correction of vitamin D deficiency through cholecalciferol supplementation is sufficient to prevent the elevations in serum PTH. However, in CKD, the recommendations of the KDIGO guidelines to correct vitamin D deficiency prior to initiating therapy with calcitriol often fails to correct serum PTH. In fact, in CKD stages 3–4, daily cholecalciferol doses of 4,000 IU/day for 1 month followed by 2,000 IU for 2 additional months, which effectively corrected serum 25(OH)D from 14 to 37 ng/ml, did not reduce serum PTH, despite achieving normal serum calcitriol levels (54). Instead, in the same CKD3-4 group, 50% of patients receiving 50,000 IU of ergocalciferol (vitamin D2) every 14 days, reached 25(OH)D levels above 35 ng/ml that suppressed PTH (5), while daily doses of 8,000 IU of cholecalciferol for 12 weeks effectively controlled SHPT without hyper-calcemic or hyper-phosphatemic episodes (55). In renal transplant recipients, effective PTH suppression requires 100,000 IU of ergocalciferol every 14 days (56).

Part of the difficulty in normalizing serum PTH with vitamin D supplementation as CKD progresses results from the impact of prolonged hypocalcemia or vitamin D deficiency on parathyroid cell proliferation to meet the requirements for higher serum PTH to normalize serum calcium. Persistent hyperphosphatemia also stimulates parathyroid hyperplasia (57).

**Figure 2:**
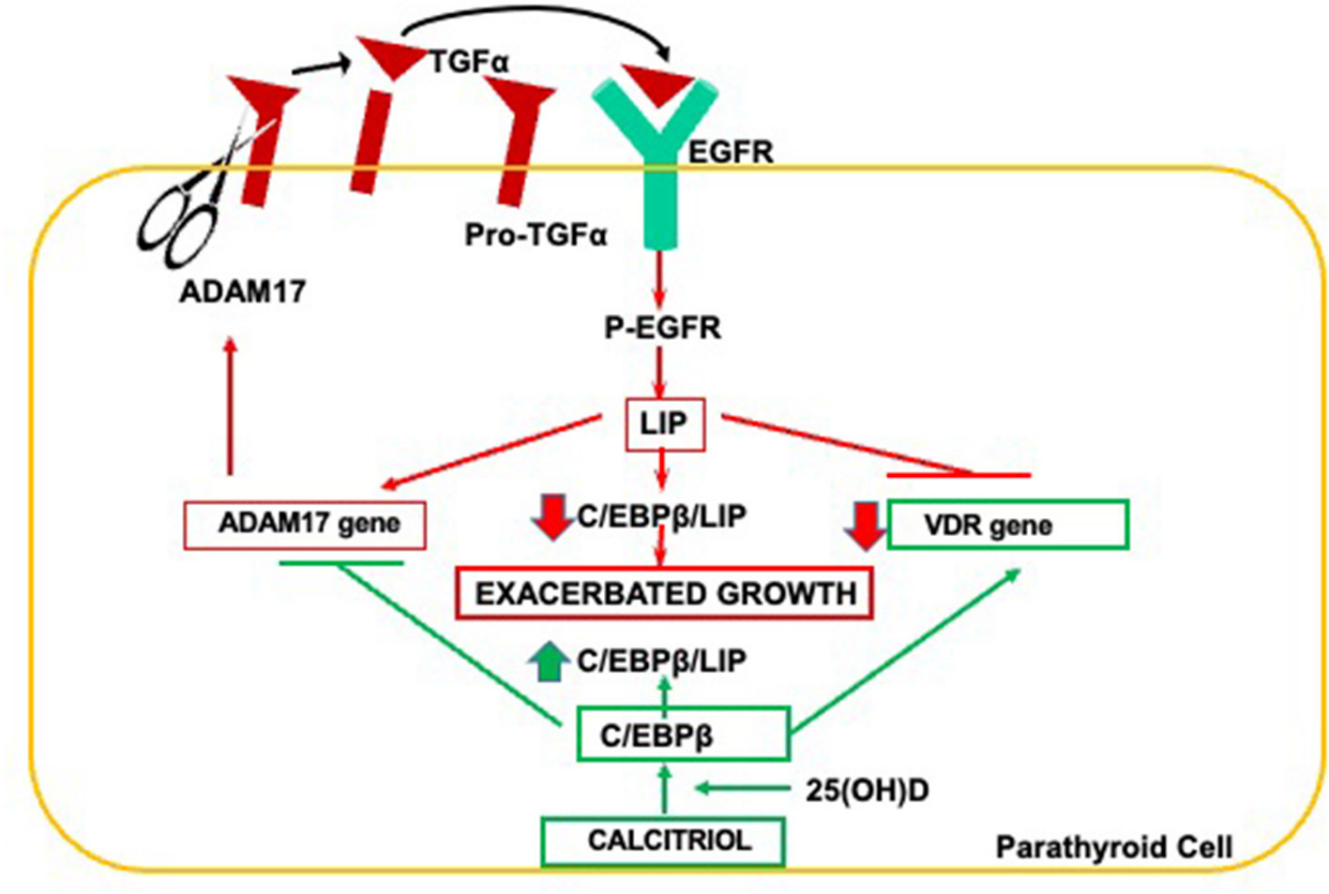
Pathogenesis of parathyroid hyperplasia and resistance to calcitriol actions in CKD. Increases in ADAM17 release of TGFα initiate a vicious ADAM17/TGFα-EGFR cycle responsible for elevations in LIP, the dominant negative isoform of C/EBPβ. Reductions in parathyroid C/EBPβ/LIP ratio induce the ADAM17 gene and suppress VDR gene expression exacerbating parathyroid growth and creating resistance to calcitriol actions. Synergistic interactions between 25(OH)D and calcitriol induce C/EBPβ expression to safely counteract both exacerbated growth and VDR reductions.

The severity of parathyroid cell growth determines not only the degree of SHPT but also contributes to marked reductions in parathyroid VDR, calcium sensing receptor, FGF receptors, and cell membrane klotho, thus impairing PTH suppression in response to the correction of vitamin D deficiency or to therapeutic interventions with calcitriol or its analogs, oral calcium, or by the progressive elevations in FGF23 that take place in the course of CKD. The pathogenic link between parathyroid hyperplasia and VDR reductions and its reversal by synergistic 25(OH)D/calcitriol interactions is summarized in Figure 2.

The release of mature TGFα from its transmembrane precursor by ADAM17, an enzyme essential for parathyroid gland development (38), initiates a powerful autocrine loop for excessive TGFα/EGFR-growth signals because TGFα induces its own gene expression (58) and that of the ADAM17 gene (38). TGFα/EGFR-driven increases of oncogenic LIP is responsible for the suppression of VDR gene expression (59) and may contribute to the transformation of parathyroid growth from diffuse to nodular (60). The importance of this pathway to the degree of SHPT and resistance to vitamin D therapy was corroborated in nephrectomized mice harboring a parathyroid-specific EGFR inactivation (35). Importantly, in a rat model of hyperphosphatemic CKD, when intraperitoneal doses of 25(OH)D that correct vitamin D deficiency but are insufficient to reduce serum PTH, are combined with a paricalcitol dose, also insufficient *per se* to suppress serum PTH or parathyroid cell growth, the 25(OH)D/paricalcitol combination effectively inhibited parathyroid ADAM17 resulting in a 50% PTH reduction (38), despite no changes in serum calcium (38). Mechanistically, improved parathyroid calcitriol synthesis upon vitamin D supplementation, and/or synergistic 25(OH)D/calcitriol interactions that enhance VDR activation partly explain the higher efficacy of the combination to inhibit serum PTH and parathyroid hyperplasia over that of either monotherapy. This synergy may help improve the control of SHPT in advanced CKD patients, whose degree of hypercalcemia or hyperphosphatemia impedes escalating calcitriol or analog dosage. Importantly, a strict control of doses is mandatory, particularly when using oral formulations, as the 25(OH)D/calcitriol (analog) combination will also synergize to activate intestinal VDR increasing calcium and phosphorus absorption. Undoubtedly, the risks of hypercalcemia and hyperphosphatemia will be lower if cholecalciferol, rather than 25(OH)D, is used to normalize vitamin D levels.

## Direct Renoprotective Actions of the Calcitriol/VDR Complex

### Induction of CYP24A1

In the kidney, the induction of CYP24A1 was considered for decades the most critical action of the calcitriol/VDR complex, as it maintains serum calcitriol within normal limits to prevent hypercalcemia and hyperphosphatemia. CYP24A1 degrades excessive circulating levels of calcitriol and also, of 25(OH)D. In agreement with the distinct calcitropic potency of these two vitamin D metabolites, CYP24A1 has a 25-fold higher affinity for calcitriol than for 25(OH)D (18). The severe hypercalcemia and nephrocalcinosis in children and adults with a loss of function mutation of this gene (61, 62), which corroborated the phenotype of the CYP24A1 null mouse (63), strongly supports the clinical significance of calcitriol induction of CYP24A1 in vitamin D responsive tissues.

### Induction of Klotho

Progressive reductions in renal content of the longevity gene klotho (64) increase mortality rates in the course of CKD due to accelerated skeletal, immune, renal and cardiovascular aging.

Klotho is expressed mostly in the kidney, the parathyroid gland, and the choroid plexus (65). Interestingly, only renal klotho content appears to be essential for survival, since a kidney specific klotho ablation sufficed to reproduce the accelerated skeletal, immune, renal and cardiovascular aging of the klotho null mice (66). This finding and the identification of a VDRE in the human klotho promoter (67) suggested that defects in vitamin D induction of renal klotho could mediate, in part, the epidemiological association between vitamin D deficiency and higher risk of all-cause mortality in the general population (68), a risk markedly augmented in CKD patients (9). In fact, the lower survival rates in hemodialysis patients carrying a klotho polymorphism that impaired function were improved by calcitriol (analog) administration (69). However, it is unclear whether the increases in survival involved elevations in the levels of the defective klotho, or direct pro-survival actions of vitamin D interventions unrelated to the induction of renal klotho.

Our understanding of the cross-talk between CKD and loss of klotho pro-survival actions, including protection against CKD progression, has evolved substantially in the last 10 years. Initially, membrane bound renal klotho was considered, exclusively, a high affinity receptor for circulating FGF23 and, therefore, essential for FGF23 phosphaturic actions that attenuate the pro-aging features of hyperphosphatemia: SHPT, systemic inflammation, vascular calcification (70).

Klotho also exists in a soluble form, with FGF23-independent endocrine actions. Soluble klotho is generated by proteolytic cleavage of the transmembrane klotho, is found in blood, urine and cerebrospinal fluid (71, 72), and is associated with identical survival benefits, which also include phosphaturic actions, as well as anti-inflammatory, anti-apoptotic, and anti-oxidant properties (73, 74).

Distinct mechanisms mediate the phosphaturic actions of membrane bound- and soluble klotho (s-klotho). The FGF23/FGFR/membrane bound klotho complex decreases the expression of the sodium-phosphate co-transporter NPT2a at the cell surface of proximal tubular cells reducing phosphate reabsorption (75). Instead, S-klotho cleaves residues in the NPT2a that promote its endocytosis, thus impeding phosphate entrance into renal tubular cells in an FGF23- independent manner (72). Clinically, the importance of the FGF23/klotho and S-klotho actions to attenuate the mortality risks associated with hyperphosphatemia were demonstrated in CKD patients stages 3–4. Progressive reductions of renal klotho impaired the response to high serum FGF23, markedly reducing fractional excretion of phosphate and causing 4-fold higher Kauppila indexes, a measure of abdominal aortic calcification (76). Calcitriol induction of renal klotho will reduce the resistance to FGF23-driven phosphaturia. In addition, calcitriol/VDR induction of renal megalin (22), essential for the endocytosis of the NPT2 co-transporter (77), could synergize with the phosphaturic actions of s-klotho.

In mice with normal kidney function, Klotho induction of autophagy by disruption of the formation of the Beclin1/Bcl2 complex (78), is one of the mechanisms critical for klotho prevention of premature aging and lifespan improvements that are unrelated to the attenuation of hyperphosphatemia. Similar Klotho-mediated increases in authophagic flow could explain why systemic administration of recombinant klotho rescues the renal and cardiovascular damage associated to acute or chronic renal injury (79–81).

Significantly, two mouse models of acute kidney injury (AKI), namely bilateral ischemia reperfusion injury, and unilateral ischemia and unilateral nephrectomy, have demonstrated that klotho/s-klotho induction of autophagic flow also contributes to attenuate AKI progression to CKD. Briefly, it is well-accepted that despite a complete recovery of renal function, AKI later progresses to CKD with decreases in creatinine clearance, hyperphosphatemia, and increases in renal fibrosis. While klotho haplo-insufficient mice progressed to CKD much faster, klotho overexpressing mice were protected. Importantly, administration of recombinant α-klotho also protected mice from AKI-driven CKD mostly through increases in renal cell autophagic flow. However, the anti-oxidant, anti-apoptotic actions of soluble klotho cannot be ruled out (82).

Vitamin D anti-oxidant, pro-autophagic, and anti-apoptotic properties may synergize with klotho actions to protect the kidney from CKD progression (83).

Overall, preventing the reduction of renal and/or circulating klotho is essential to reduce the severity of CKD progression and the risk for cardiovascular mortality. Indeed, serum s-klotho decreases with age, hypertension (84), and systemic inflammation (85), all recognized determinants of renal damage and cardiovascular disease.

The main contribution of the kidney to serum s-klotho levels was strongly supported by an 80% reduction in circulating s-klotho upon specific ablation of renal klotho (66). This finding was critical to consider that serum s-klotho levels could be an accurate biomarker of renal klotho content, CKD progression and of cardiovascular mortality risk in CKD patients. However, the kidney is also the main organ for the clearance of circulating klotho into the urine, through a process of transcytosis through tubular cells (86). Therefore, increases in circulating s-klotho could reflect an impaired transcytosis to the urine by the damaged kidney, which may mask both, actual renal klotho reductions and potential improvements in renal klotho content induced by vitamin D interventions. Therefore, before serum or urinary s- klotho can be used as biomarkers of renal and cardiovascular risk, it will be important to establish the optimal range for serum and urinary s-klotho that are associated with the lowest mortality risks at different CKD stages.

Regarding therapy with calcitriol or its analogs to recover renal klotho in advanced CKD, it is important to consider the severe alterations in the vitamin D/klotho-FGF23 axis in CKD to avoid hyperphosphatemia and excess levels of circulating calcitriol that could compromise klotho pro-survival actions.

## Calcitriol Reduction of Hypertension-Driven Renal and Vascular Damage Unrelated to the Suppression of the Renin Gene

### Suppression of Renin

Vitamin D deficiency has been associated with the development of hypertension. Calcitriol/VDR suppression of renin gene expression (87) explains in part the causal association between increases in circulating 25(OH)D levels and reductions in blood pressure and hypertension demonstrated by Mendelian randomization analysis (88). However, randomized controlled trials in individuals with normal renal function have yielded controversial results, partly due to variable vitamin D interventions as well as the inclusion of subjects with normal vitamin D levels or receiving anti-hypertensive medications (89). In experimental models of diabetic nephropathy, the simultaneous administration of the angiotensin receptor 1 (AT1R) inhibitor Losartan and the calcitriol analog paricalcitol effectively attenuated Losartan-induced compensatory increases in renin, which resulted in much lower serum angiotensin II (90). The efficacy of calcitriol (analog) therapy at decreasing the activation of the RAAS has also demonstrated a beneficial impact on proteinuria, systemic inflammation and the progression of the cardiorenal syndrome (91). However, as will be presented below, the calcitriol (analog)/VDR complex may ameliorate angiotensin II–driven renal damage independently of its downregulation of RAAS activation by suppressing renin gene expression.

### Suppression of ADAM17

**Figure 3:**
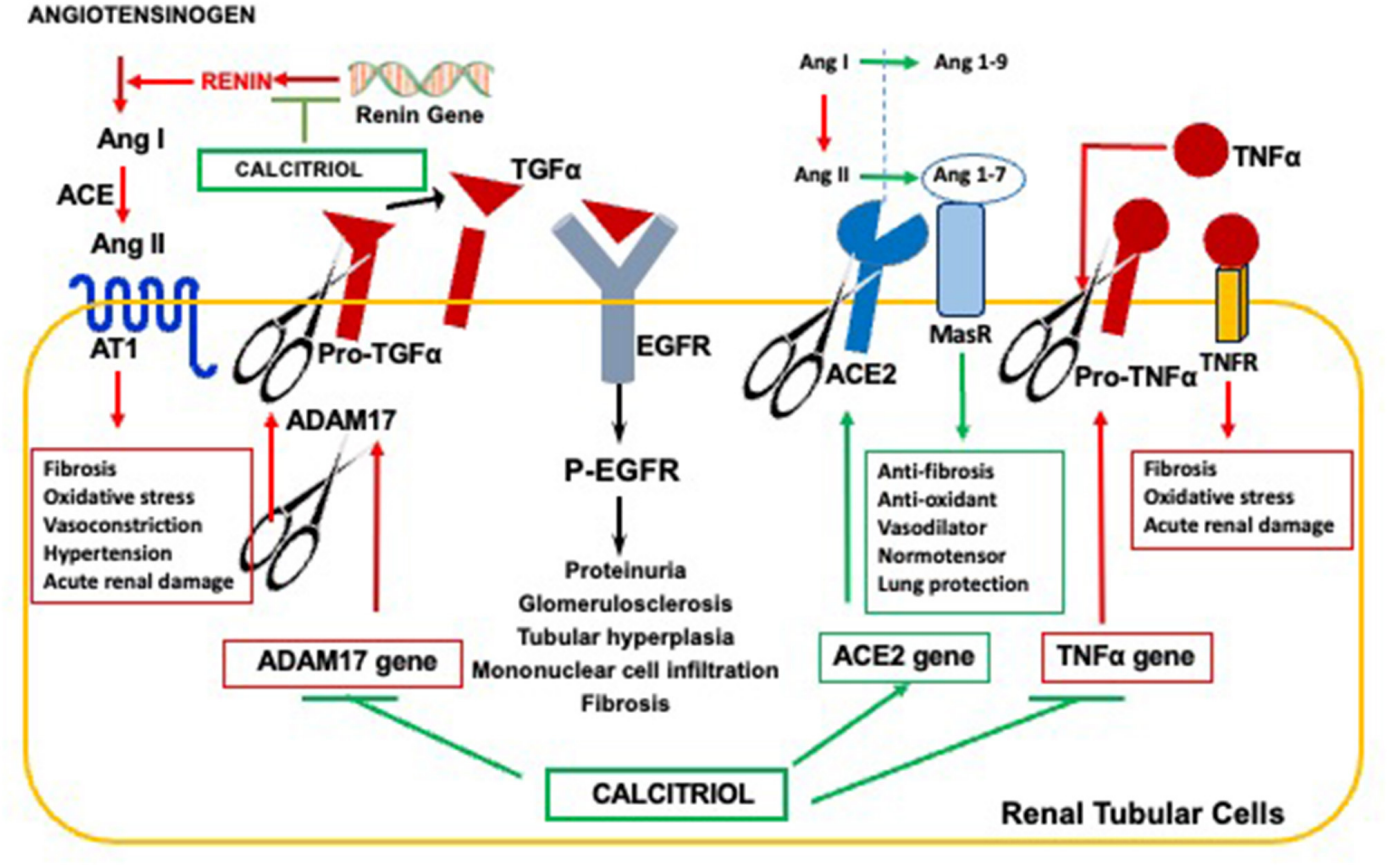
Anti-hypertensive properties of the calcitriol-VDR complex reducing CKD Progression. Calcitriol ameliorates hypertension-driven renal damage by suppression of renin gene expression, responsible for elevations in circulating Angiotensin II; Inhibition of ADAM17 expression to effectively counteract Angiotensin II-mediated increases in TGFα/EGFR signals responsible for CKD progression; Induction of ACE2 for Angiotensin 1-7 synthesis and Angiotenisn 1-7/MAS receptor anti-hypertensive, anti-fibrotic and anti-inflammatory signals, and Inhibition of TNFα expression, whose induction of the ADAM17 gene further augments systemic inflammation and renal injury.

The pioneer work of Lautrette and collaborators demonstrated that Angiotensin II causes tubular hyperplasia, fibrosis, glomerulosclerosis, proteinuria, and inflammatory infiltration to the renal parenchyma through the enhancement of ADAM17 activity at the surface of renal tubular cells (Summarized in Figure 3) (37). Significantly, ADAM17-promoted release of TGFα to drive EGFR activation—the pathway responsible for the onset of parathyroid hyperplasia and resistance to calcitriol/VDR actions—also mediates angiotensin-driven progression of renal injury. In fact, in a mouse model of mild CKD, either ablation of TGFα, inhibition of EGFR activation, or inhibition of ADAM17 markedly reduced the degree of renal injury in response to a prolonged exposure to high levels of angiotensin II, despite persistent hypertension.

Since the activation of renal ADAM17/TGFα signals occurs in human CKD of any etiology (92), calcitriol/VDR suppression of ADAM17 (38) could contribute to ameliorate CKD progression in hypertensive individuals (see Figure 3). The combined suppression of renin and ADAM17 gene expression by calcitriol could contribute to the synergy between paricalcitol and the ACE-inhibitor, enalapril. In addition, the increases in renal ADAM17 in CKD also contribute to the renal damage caused by excessive inflammation.

ADAM17, also called TACE for Tumor Necrosis Factor Converting Enzyme, is the enzyme responsible for the release of TNFα to the circulation. Since TNFα induces ADAM17 gene transcription (93), a vicious cycle is initiated to increase renal ADAM17 and TNFα-driven systemic inflammation, further potentiating renal inflammatory damage in an angiotensin II-independent manner. Consequently, this process is no longer responsive to anti-RAS therapy. Calcitriol/VDR suppression of renin, ADAM17 and, also of TNFα gene expression, explain the synergy between the calcitriol analog paricalcitol and the angiotensin II converting enzyme inhibitor enalapril at reducing inflammatory macrophage infiltration in the renal parenchyma in rat CKD (94).

### Induction of ACE2

Calcitriol induction of Angiotensin converting enzyme II (ACE2) expression can effectively counteract angiotensin II-driven hypertensive, inflammatory and pro-fibrotic signals, as depicted in Figure 3. ACE2 catalyzes the conversion of both angiotensin I and angiotensin II into angiotensin 1-9 and angiotensin 1-7, respectively, thereby reducing circulating levels and the deleterious effects of an excess of angiotensin II that follows RAS hyperactivation (95). In addition to reducing circulating angiotensin II, angiotensin 1-7 binding to its receptor MAS activates signaling pathways that promote anti-fibrotic, anti-oxidant, and vasodilatory signals, thus favoring multiorgan protection and normotension (96, 97).

Calcitriol induction of ACE2 exerts neuroprotective effects in the hypertensive brain by attenuating ROS production and shifting microglia polarization from M1 to M2 (98), and also prevents LPS-induced acute lung injury by attenuating the accelerated neutrophil infiltration and severe inflammation of the lung that follows ACE2 reductions (99). However, in CKD patients with no history of cardiovascular damage, levels of circulating, soluble ACE2 correlated with the classical cardiovascular risk factors (older age, diabetes, male gender) (100). Since ADAM17 cleaves ACE2, the increases in ADAM17 expression and activity that occurs in kidney disease of all etiologies could partly account for the increases in circulating ACE2 and a higher risk for cardiovascular events (100). Conversely, the cardiovascular protection by calcitriol and its analogs could result from suppression of ADAM17 expression and induction of ACE2. In fact, in non-obese diabetic mice, the calcitriol analog paricalcitol, alone or in combination with aliskiren, effectively counteracted the rise in circulating, soluble ACE2 levels associated with diabetes. Reduced ADAM17 expression, oxidative stress and proteinuria (101) were also observed, emphasizing the reno-protective effects of ACE2 induction.

In summary, the actions of the vitamin D endocrine system at mitigating RAS hyperactivation extend far beyond the initial discovery of calcitriol's ability to suppress renin gene expression. Calcitriol exerts reno-protective effects to attenuate CKD progression induced by excessive angiotensin II by (1) counteracting ADAM17/TGFα signals, (2) inducing ACE2/Angiotensin 1-7/MAS receptor activity at the cell surface by preventing ACE2 shedding in the setting of increased ADAM17 activity in uremia and, (3) by preventing excessive renal inflammation through the simultaneous suppression of the vicious TNFα/ADAM17 feed-forward loop. Undoubtedly, in the course of CKD, elevations in circulating ACE2 activity provide a novel biomarker of the failure of vitamin D/calcitriol interventions to effectively suppress the deleterious effects of ADAM17 and RAS hyperactivation.

Despite the recommendation that circulating levels of 25(OH)D above 23 ng/ml may effectively attenuate CKD progression (10), it is imperative to recognize that levels above 35 ng/ml may be necessary to suppress PTH with cholecalciferol supplementation in more advanced CKD, as angiotensin II-driven increases in ADAM17/TGFα signaling may cause renal resistance to calcitriol actions through VDR reductions, as demonstrated in hyperplastic parathyroid glands. Thus, optimal vitamin D intervention may require the synergistic 25(OH)D/calcitriol (analog) interactions through normalization of vitamin D levels and low doses of calcitriol (analogs).

## Calcitriol Attenuation of Immune Cell Function Augmenting Systemic Inflammation and Oxidative Stress-Mediated Multi-Organ Damage

Calcitriol is a well-recognized immunomodulator. A normal vitamin D status is essential for adequate function of the immune system to ensure protection from bacterial and viral infections, minimize progression of autoimmune disorders, and avoid excessive inflammation (102). The benefits of normal vitamin D on innate and acquired immunity involve multiple mechanisms including decreasing the antigenicity of antigen presenting cells, downregulating the production of pro-fibrotic and pro-inflammatory Th1 cytokines, and enhancing the production of anti-inflammatory Th2 cytokines. Calcitriol/VDR acts on T cells to shift their polarization toward a Th2/regulatory phenotype and stimulates T regulatory lymphocyte development (1, 102). Calcitriol inhibition of Skp2 expression is a key mechanism for the maintenance of T regs (103) and may be critical at avoiding the blockage on autophagic flow driven by the cell invasion by Middle East respiratory syndrome coronavirus (MERS-CoV) (104). Calcitriol immune regulatory properties confer protection not only from inflammation-driven multiple organ damage but also from inflammation-driven reductions of renal klotho that accelerate CKD progression.

ADAM17-mediated release of TNFα to the circulation is a main determinant of the degree of systemic inflammation and renal and cardiovascular damage. In fact, a polymorphism of ADAM17 that results in a mild increase in TNFα release is associated with a higher degree of cardiovascular mortality in individuals with normal renal function (105). Furthermore, specific deletion of ADAM17 in myeloid cells markedly reduces mortality rates in a rat model of LPS-induced sepsis (106).

In CKD patients, circulating mononuclear cells express higher levels of ADAM17 and higher serum levels of TNFα, which can be reduced by combined vitamin D and calcitriol (analog) administration (107). These circulating mononuclear cells from hemodialysis patients also have a 40% reduced capacity for 25(OH)D uptake, which mimics impaired 25(OH)D uptake in renal proximal tubular cells with advancing CKD. Interestingly, even when mononuclear cells do not express megalin, the defective uptake was corrected with calcitriol administration (108), further supporting the use of combined 25(OH)D and calcitriol (analogs) as discussed with hyperplastic parathyroid cells.

In the vasculature, calcitriol suppression of the ADAM17/TNFα loop should decrease TNFα-induction of vascular neutral sphyngomyelinase 2 that drives the release of pro-calcifying exosomes propagating—calcium deposition (109). Moreover, calcitriol induction of miR145 is critical for vascular health, as this is the prevalent miR in vascular smooth muscle cells and the master regulator of their contractile phenotype (110). Reductions in miR145 are associated with vascular diseases and calcification (111). Importantly, decreases in miR145 increase ADAM17 gene expression (112), thus initiating the ADAM17-TNFα loop for vascular injury. It is unclear at present whether monocyte expression of ADAM17, TNFα and neutral sphingomyelinase 2 reflect their vascular levels. Therefore, the assessment of the benefits of vitamin D/calcitriol interventions on vascular health in the course of CKD is limited to less sensitive markers of changes in vascular function, such as pulse wave velocity or Kauppila index. Finally, as indicated earlier, the induction of ADAM17 by increased angiotensin II provides a causal link between hypertension and inflammation. Importantly, as described below, a novel causal link between inflammation driving hypertension has been identified recently.

## Inflammation-Induced Renin-Driven Hypertension

Hypertension and inflammation are interrelated processes. In mice, while the absence of monocyte lineage prevents angiotensin II-driven hypertension (113), the overactivation of the renin-angiotensin system results in renal and vascular accumulation of proinflammatory macrophages resulting in increased oxidative stress and its associated tissue damage. In the vasculature, hypertension-induced macrophage infiltration drives nitric oxide scavenging causing reductions in renal blood flow (114), which in turn induce renin secretion by juxtaglomerular cells (114). However, until recently, there was no evidence for immune cell-induced hypertension or its modulation by vitamin D. The pioneer work by Oh and collaborators has demonstrated that increases in ER stress in response to vitamin D deficiency are sufficient to cause renin dependent hypertension through the secretion of micro-RNA 106b-5p that enables a direct communication between innate immune cells and juxtaglomerular cells (115). Importantly, the miR106b-5p link between inflammatory immune cells and renin secretion by juxtaglomerular cells can be prevented by the calcitriol/VDR complex.

**Figure 4:**
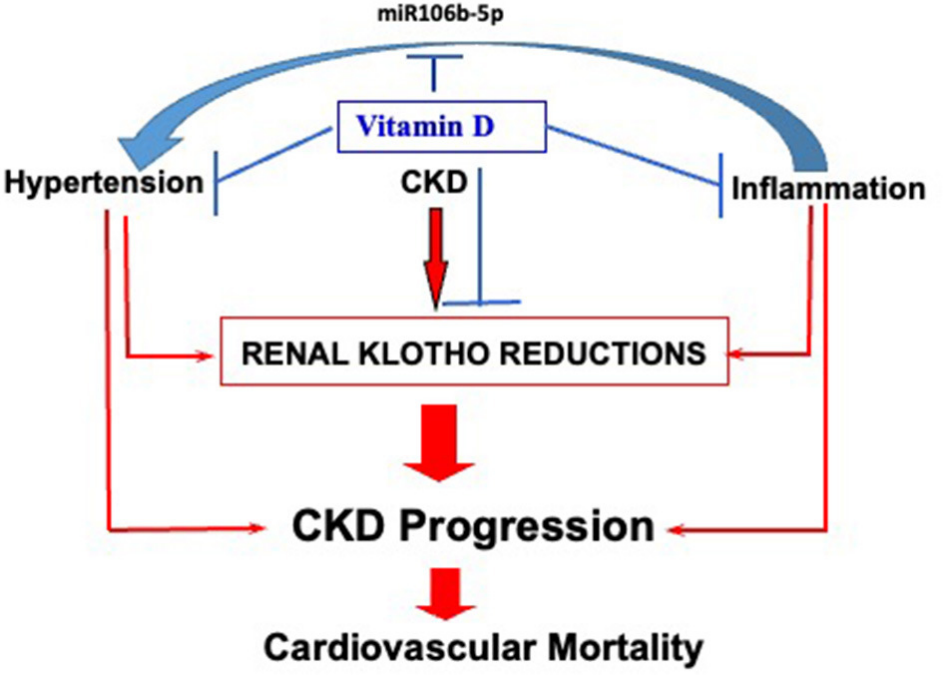
Integration of non-classical vitamin D actions conferring renoprotection. Vitamin D protects against CKD progression through direct inhibition of systemic inflammation, hypertension and renal klotho reductions; indirect attenuation of the reductions in renal klotho content through the control of its most potent downregulators, systemic inflammation and hypertension; inhibition of the release of miR106b-5p by pro-inflammatory monocyte-macrophages, a novel link between inflammation with renin-dependent hypertension.

In summary, as depicted in Figure 4, the integrity of the vitamin D endocrine system protects from CKD progression through direct inhibition of renal klotho reductions, systemic inflammation, and hypertension. Maintenance of renal klotho ensures the reduction of the pro-aging actions of phosphate retention, as well as the longevity properties of klotho-mediated induction of renal autophagic flow. However, circulating soluble klotho is not an accurate marker of renal klotho reductions.

Vitamin D-mediated suppression of hypertension has a dual impact on CKD progression by reducing angiotensin II-driven renal damage and attenuating the reductions in renal klotho. The former is achieved through the maintenance of an adequate balance between pro-hypertensive (Renin and ADAM17) and anti-hypertensive signals (ACE2). Increases in circulating ACE2 levels provide an accurate measurement of increased ADAM17 activity and the cardiovascular risk associated to reductions in ACE2 expression at the cell membrane in CKD, which may be corrected with calcitriol (analog) therapy. Finally, calcitriol reduction of miR106b-5p release to the circulation by pro-inflammatory monocyte-macrophages provides the first causal link between macrophage ER stress activation and the induction of renin-dependent hypertension.

At this time, the optimal supplementation strategy to ensure the desired outcome is not known. This is due to a tremendous variability among individuals in their capacity non only for local calcitriol production, but also for the local bioactivation of vitamin D to 25(OH)D in tissues bearing 25-hydroxylases. To overcome these limitations, it is mandatory to examine whether miR106b, circulating ACE2, angiotensin II, or angiotensin 1-7 levels can better reflect the benefits of vitamin D/calcitriol (analog) interventions and serve as biomarkers to optimize the reno-protective effects of vitamin D therapy in CKD compared to circulating 25(OH)D levels.

## Author Contributions

AD, KB, and CB-M wrote sections of the manuscript. All authors contributed to manuscript revision, read, and approved the submitted version.

## Funding

This work was supported by NIH RO1HL09481806 and VA Merit Award 1BX003648-01 and P30DK020579.

## Conflict of Interest

The authors declare that the research was conducted in the absence of any commercial or financial relationships that could be construed as a potential conflict of interest.

## Publisher's Note

All claims expressed in this article are solely those of the authors and do not necessarily represent those of their affiliated organizations, or those of the publisher, the editors and the reviewers. Any product that may be evaluated in this article, or claim that may be made by its manufacturer, is not guaranteed or endorsed by the publisher.

## References

[1] Adams JS Hewison M. Update in vitamin D. J Clin Endocrinol Metab. (2010) 95:471–8. 10.1210/jc.2009-1773 20133466 PMC2840860

[2] Chowdhury R Kunutsor S Vitezova A Oliver-Williams C Chowdhury S Kiefte-de-Jong JC. Vitamin D and risk of cause specific death: systematic review and meta-analysis of observational cohort and randomised intervention studies. BMJ. (2014) 348:g1903. 10.1136/bmj.g1903 24690623 PMC3972416

[3] Ortiz A Covic A Fliser D Fouque D Goldsmith D Kanbay M. Epidemiology, contributors to, and clinical trials of mortality risk in chronic kidney failure. Lancet. (2014) 383:1831–43. 10.1016/S0140-6736(14)60384-6 24856028

[4] London GM Guerin AP Marchais SJ Metivier F Pannier B Adda H. Arterial media calcification in end-stage renal disease: impact on all-cause and cardiovascular mortality. Nephrol Dial Transplant. (2003) 18:1731–40. 10.1093/ndt/gfg414 12937218

[5] Al-Badr W Martin KJ. Vitamin D and kidney disease. Clin J Am Soc Nephrol. (2008) 3:1555–60. 10.2215/CJN.01150308 18450926 PMC4571143

[6] Ellam TJ Chico TJ. Phosphate: the new cholesterol? The role of the phosphate axis in non-uremic vascular disease. Atherosclerosis. (2012) 220:310–8. 10.1016/j.atherosclerosis.2011.09.002 21962238

[7] Hu MC Kuro-o M Moe OW. The emerging role of Klotho in clinical nephrology. Nephrol Dial Transplant. (2012) 27:2650–7. 10.1093/ndt/gfs160 22802580 PMC3398064

[8] LaClair RE Hellman RN Karp SL Kraus M Ofner S Li Q. Prevalence of calcidiol deficiency in CKD: a cross-sectional study across latitudes in the United States. Am J Kidney Dis. (2005) 45:1026–33. 10.1053/j.ajkd.2005.02.029 15957131

[9] Melamed ML Astor B Michos ED Hostetter TH Powe NR Muntner P. 25-hydroxyvitamin D levels, race, and the progression of kidney disease. J Am Soc Nephrol. (2009) 20:2631–9. 10.1681/ASN.2009030283 19875805 PMC2794237

[10] Nakano C Hamano T Fujii N Matsui I Tomida K Mikami S. Combined use of vitamin D status and FGF23 for risk stratification of renal outcome. Clin J Am Soc Nephrol. (2012) 7:810–9. 10.2215/CJN.08680811 22362065

[11] Dusso AS Brown AJ Slatopolsky E. Vitamin D. Am J Physiol Renal Physiol. (2005) 289:F8–28. 10.1152/ajprenal.00336.2004 15951480

[12] Webb AR Kline L Holick MF. Influence of season and latitude on the cutaneous synthesis of vitamin D3: exposure to winter sunlight in Boston and Edmonton will not promote vitamin D3 synthesis in human skin. J Clin Endocrinol Metab. (1988) 67:373–8. 10.1210/jcem-67-2-373 2839537

[13] Cheng JB Levine MA Bell NH Mangelsdorf DJ Russell DW. Genetic evidence that the human CYP2R1 enzyme is a key vitamin D 25-hydroxylase. Proc Natl Acad Sci USA. (2004) 101:7711–5. 10.1073/pnas.0402490101 15128933 PMC419671

[14] Brown AJ Ritter CS Knutson JC Strugnell SA. The vitamin D prodrugs 1alpha(OH)D2, 1alpha(OH)D3 and BCI-210 suppress PTH secretion by bovine parathyroid cells. Nephrol Dial Transplant. (2006) 21:644–50. 10.1093/ndt/gfi186 16221703

[15] Bergada L Pallares J Maria Vittoria A Cardus A Santacana M Valls J. Role of local bioactivation of vitamin D by CYP27A1 and CYP2R1 in the control of cell growth in normal endometrium and endometrial carcinoma. Lab Invest. (2014) 94:608–22. 10.1038/labinvest.2014.57 24732451

[16] Heaney RP Armas LA Shary JR Bell NH Binkley N Hollis BW. 25-Hydroxylation of vitamin D3: relation to circulating vitamin D3 under various input conditions. Am J Clin Nutr. (2008) 87:1738–42. 10.1093/ajcn/87.6.1738 18541563

[17] Dusso AS Tokumoto M. Defective renal maintenance of the vitamin D endocrine system impairs vitamin D renoprotection: a downward spiral in kidney disease. Kidney Int. (2011) 79:715–29. 10.1038/ki.2010.543 21270766

[18] Haussler MR Whitfield GK Kaneko I Haussler CA Hsieh D Hsieh JC. Molecular mechanisms of vitamin D action. Calcif Tissue Int. (2013) 92:77–98. 10.1007/s00223-012-9619-0 22782502

[19] Jacob AI Sallman A Santiz Z Hollis BW. Defective photoproduction of cholecalciferol in normal and uremic humans. J Nutr. (1984) 114:1313–9. 10.1093/jn/114.7.1313 6330321

[20] Nykjaer A Dragun D Walther D Vorum H Jacobsen C Herz J. An endocytic pathway essential for renal uptake and activation of the steroid 25-(OH) vitamin D3. Cell. (1999) 96:507–15. 10.1016/S0092-8674(00)80655-8 10052453

[21] Takemoto F Shinki T Yokoyama K Inokami T Hara S Yamada A. Gene expression of vitamin D hydroxylase and megalin in the remnant kidney of nephrectomized rats. Kidney Int. (2003) 64:414–20. 10.1046/j.1523-1755.2003.00114.x 12846736

[22] Liu W Yu WR Carling T Juhlin C Rastad J Ridefelt P. Regulation of gp330/megalin expression by vitamins A and D. Eur J Clin Invest. (1998) 28:100–7. 10.1046/j.1365-2362.1998.00253.x 9541123

[23] Halloran BP Schaefer P Lifschitz M Levens M Goldsmith RS. Plasma vitamin D metabolite concentrations in chronic renal failure: effect of oral administration of 25-hydroxyvitamin D3. J Clin Endocrinol Metab. (1984) 59:1063–9. 10.1210/jcem-59-6-1063 6333430

[24] Dusso A Lopez-Hilker S Rapp N Slatopolsky E. Extra-renal production of calcitriol in chronic renal failure. Kidney Int. (1988) 34:368–75. 10.1038/ki.1988.190 3172645

[25] Krajisnik T Bjorklund P Marsell R Ljunggren O Akerstrom G Jonsson KB. Fibroblast growth factor-23 regulates parathyroid hormone and 1alpha-hydroxylase expression in cultured bovine parathyroid cells. J Endocrinol. (2007) 195:125–31. 10.1677/JOE-07-0267 17911404

[26] Dai B David V Alshayeb HM Showkat A Gyamlani G Horst RL. Assessment of 24,25(OH)2D levels does not support FGF23-mediated catabolism of vitamin D metabolites. Kidney Int. (2012) 82:1061–70. 10.1038/ki.2012.222 22739976 PMC3461248

[27] Bosworth CR Levin G Robinson-Cohen C Hoofnagle AN Ruzinski J Young B. The serum 24,25-dihydroxyvitamin D concentration, a marker of vitamin D catabolism, is reduced in chronic kidney disease. Kidney Int. (2012) 82:693–700. 10.1038/ki.2012.193 22648296 PMC3434313

[28] Saramaki A Diermeier S Kellner R Laitinen H Vaisanen S Carlberg C. Cyclical chromatin looping and transcription factor association on the regulatory regions of the p21 (CDKN1A) gene in response to 1alpha,25-dihydroxyvitamin D3. J Biol Chem. (2009) 284:8073–82. 10.1074/jbc.M808090200 19122196 PMC2658101

[29] Kim S Yamazaki M Zella LA Shevde NK Pike JW. Activation of receptor activator of NF-kappaB ligand gene expression by 1,25-dihydroxyvitamin D3 is mediated through multiple long-range enhancers. Mol Cell Biol. (2006) 26:6469–86. 10.1128/MCB.00353-06 16914732 PMC1592822

[30] Centeno PP Herberger A Mun HC Tu C Nemeth EF Chang W. Phosphate acts directly on the calcium-sensing receptor to stimulate parathyroid hormone secretion. Nat Commun. (2019) 10:4693. 10.1038/s41467-019-12399-9 31619668 PMC6795806

[31] Giangreco AA Nonn L. The sum of many small changes: microRNAs are specifically and potentially globally altered by vitamin D3 metabolites. J Steroid Biochem Mol Biol. (2013) 136:86–93. 10.1016/j.jsbmb.2013.01.001 23333596 PMC3686905

[32] Liu X Cheng Y Yang J Qin S Chen X Tang X. Flank sequences of miR-145/143 and their aberrant expression in vascular disease: mechanism and therapeutic application. J Am Heart Assoc. (2013) 2:e000407. 10.1161/JAHA.113.000407 24166492 PMC3886745

[33] Taibi F Metzinger-Le Meuth V M'Baya-Moutoula E Djelouat M Louvet L Bugnicourt JM. Possible involvement of microRNAs in vascular damage in experimental chronic kidney disease. Biochim Biophys Acta. (2014) 1842:88–98. 10.1016/j.bbadis.2013.10.005 24140891

[34] Rangrez AY M'Baya-Moutoula E Metzinger-Le Meuth V Henaut L Djelouat MS Benchitrit J. Inorganic phosphate accelerates the migration of vascular smooth muscle cells: evidence for the involvement of miR-223. PLoS ONE. (2012) 7:e47807. 10.1371/journal.pone.0047807 23094093 PMC3475714

[35] Arcidiacono MV Yang J Fernandez E Dusso A. Parathyroid-specific epidermal growth factor-receptor inactivation prevents uremia-induced parathyroid hyperplasia in mice. Nephrol Dial Transplant. (2015) 30:434–40. 10.1093/ndt/gfu318 25324357 PMC4339687

[36] Gooz M. ADAM-17: the enzyme that does it all. Crit Rev Biochem Mol Biol. (2010) 45:146–69. 10.3109/10409231003628015 20184396 PMC2841225

[37] Lautrette A Li S Alili R Sunnarborg SW Burtin M Lee DC. Angiotensin II and EGF receptor cross-talk in chronic kidney diseases: a new therapeutic approach. Nat Med. (2005) 11:867–74. 10.1038/nm1275 16041383

[38] Arcidiacono MV Yang J Fernandez E Dusso A. The induction of C/EBPbeta contributes to vitamin D inhibition of ADAM17 expression and parathyroid hyperplasia in kidney disease. Nephrol Dial Transplant. (2015) 30:423–33. 10.1093/ndt/gfu311 25294851 PMC4339686

[39] Haussler MR Whitfield GK Kaneko I Forster R Saini R Hsieh JC. The role of vitamin D in the FGF23, klotho, and phosphate bone-kidney endocrine axis. Rev Endocr Metab Disord. (2012) 13:57–69. 10.1007/s11154-011-9199-8 21932165 PMC3288475

[40] Dusso AS. Vitamin D receptor: mechanisms for vitamin D resistance in renal failure. Kidney Int Suppl. (2003) S6–9. 10.1046/j.1523-1755.63.s85.3.x 12753256

[41] Dusso AS Negrea L Gunawardhana S Lopez-Hilker S Finch J Mori T. On the mechanisms for the selective action of vitamin D analogs. Endocrinology. (1991) 128:1687–92. 10.1210/endo-128-4-1687 2004595

[42] Teng M Wolf M Ofsthun MN Lazarus JM Hernan MA Camargo CA. Activated injectable vitamin d and hemodialysis survival: a historical cohort study. J Am Soc Nephrol. (2005) 16:1115–25. 10.1681/ASN.2004070573 15728786

[43] Cozzolino M Ketteler M Zehnder D. The vitamin D system: a crosstalk between the heart and kidney. Eur J Heart Fail. (2010) 12:1031–41. 10.1093/eurjhf/hfq112 20605845

[44] Hoenderop JG Chon H Gkika D Bluyssen HA Holstege FC St-Arnaud R. Regulation of gene expression by dietary Ca2+ in kidneys of 25-hydroxyvitamin D3-1 alpha-hydroxylase knockout mice. Kidney Int. (2004) 65:531–9. 10.1111/j.1523-1755.2004.00402.x 14717923

[45] Lou YR Molnár F Peräkylä M Qiao S Kalueff AV St-Arnaud R. 25-Hydroxyvitamin D(3) is an agonistic vitamin D receptor ligand. J Steroid Biochem Mol Biol. (2010) 118:162–70. 10.1016/j.jsbmb.2009.11.011 19944755

[46] Munetsuna E Nakabayashi S Kawanami R Yasuda K Ohta M Arai MA. Mechanism of the anti-proliferative action of 25-hydroxy-19-nor-vitamin D(3) in human prostate cells. J Mol Endocrinol. (2011) 47:209–18. 10.1530/JME-11-0008 21693624

[47] Alfieri C Ruzhytska O Vettoretti S Caldiroli L Cozzolino M Messa P. Native Hypovitaminosis D in CKD patients: from experimental evidence to clinical practice. Nutrients. (2019) 11:1918. 10.3390/nu11081918 31443249 PMC6723756

[48] Fernandez-Martin JL Martinez-Camblor P Dionisi MP Floege J Ketteler M London G. Improvement of mineral and bone metabolism markers is associated with better survival in haemodialysis patients: the COSMOS study. Nephrol Dial Transplant. (2015) 30:1542–51. 10.1093/ndt/gfv099 25920921

[49] Slatopolsky E Brown A Dusso A. Role of phosphorus in the pathogenesis of secondary hyperparathyroidism. Am J Kidney Dis. (2001) 37:S54–7. 10.1053/ajkd.2001.20740 11158862

[50] Brown AJ Zhong M Finch J Ritter C McCracken R Morrissey J. Rat calcium-sensing receptor is regulated by vitamin D but not by calcium. Am J Physiol. (1996) 270:F454–60. 10.1152/ajprenal.1996.270.3.F454 8780248

[51] Kifor O Moore FD Jr Wang P Goldstein M Vassilev P Kifor I. Reduced immunostaining for the extracellular Ca2+-sensing receptor in primary and uremic secondary hyperparathyroidism. J Clin Endocrinol Metab. (1996) 81:1598–606. 10.1210/jcem.81.4.8636374 8636374

[52] Canaff L Hendy GN. Human calcium-sensing receptor gene. Vitamin D response elements in promoters P1 P2 confer transcriptional responsiveness to 1,25-dihydroxyvitamin D. J Biol Chem. (2002) 277:30337–50. 10.1074/jbc.M201804200 12036954

[53] Canalejo R Canalejo A Martinez-Moreno JM Rodriguez-Ortiz ME Estepa JC Mendoza FJ. FGF23 fails to inhibit uremic parathyroid glands. J Am Soc Nephrol. 21:1125–35. 10.1681/ASN.2009040427 20431039 PMC3152229

[54] Moe SM Saifullah A LaClair RE Usman SA Yu Z. A randomized trial of cholecalciferol versus doxercalciferol for lowering parathyroid hormone in chronic kidney disease. Clin J Am Soc Nephrol. (2010) 5:299–306. 10.2215/CJN.07131009 20056760 PMC2827596

[55] Westerberg PA Sterner G Ljunggren O Isaksson E Elvarson F Dezfoolian H. High doses of cholecalciferol alleviate the progression of hyperparathyroidism in patients with CKD Stages 3-4: results of a 12-week double-blind, randomized, controlled study. Nephrol Dial Transplant. (2018) 33:466–71. 10.1093/ndt/gfx059 29156056 PMC6018863

[56] Courbebaisse M Thervet E Souberbielle JC Zuber J Eladari D Martinez F. Effects of vitamin D supplementation on the calcium-phosphate balance in renal transplant patients. Kidney Int. (2009) 75:646–51. 10.1038/ki.2008.549 18923386

[57] Slatopolsky E Finch J Denda M Ritter C Zhong M Dusso A. Phosphorus restriction prevents parathyroid gland growth. High phosphorus directly stimulates PTH secretion *in vitro*. J Clin Invest. (1996) 97:2534–40. 10.1172/JCI118701 8647946 PMC507339

[58] Arcidiacono MV Cozzolino M Spiegel N Tokumoto M Yang J Lu Y. Activator Protein 2{alpha} Mediates Parathyroid TGF-{alpha} Self-Induction in Secondary Hyperparathyroidism. J Am Soc Nephrol. (2008) 19:1919–28. 10.1681/ASN.2007111216 18579641 PMC2551566

[59] Arcidiacono MV Sato T Alvarez-Hernandez D Yang J Tokumoto M Gonzalez-Suarez I. EGFR activation increases parathyroid hyperplasia and calcitriol resistance in kidney disease. J Am Soc Nephrol. (2008) 19:310–20. 10.1681/ASN.2007040406 18216322 PMC2396751

[60] Zahnow CA Cardiff RD Laucirica R Medina D Rosen JM. A role for CCAAT/enhancer binding protein beta-liver-enriched inhibitory protein in mammary epithelial cell proliferation. Cancer Res. (2001) 61:261–9. 11196172

[61] Jacobs TP Kaufman M Jones G Kumar R Schlingmann KP Shapses S. A lifetime of hypercalcemia and hypercalciuria, finally explained. J Clin Endocrinol Metab. (2014) 99:708–12. 10.1210/jc.2013-3802 24423361 PMC3942238

[62] Schlingmann KP Kaufmann M Weber S Irwin A Goos C John U. Mutations in CYP24A1 and idiopathic infantile hypercalcemia. N Engl J Med. (2011) 365:410–21. 10.1056/NEJMoa1103864 21675912

[63] St-Arnaud R Arabian A Travers R Barletta F Raval-Pandya M Chapin K. Deficient mineralization of intramembranous bone in vitamin D-24- hydroxylase-ablated mice is due to elevated 1,25-dihydroxyvitamin D and not to the absence of 24,25-dihydroxyvitamin D. Endocrinology. (2000) 141:2658–66. 10.1210/endo.141.7.7579 10875271

[64] Kuro-o M Matsumura Y Aizawa H Kawaguchi H Suga T Utsugi T. Mutation of the mouse klotho gene leads to a syndrome resembling ageing. Nature. (1997) 390:45–51. 10.1038/36285 9363890

[65] Li SA Watanabe M Yamada H Nagai A Kinuta M Takei K. Immunohistochemical localization of Klotho protein in brain, kidney, and reproductive organs of mice. Cell Struct Funct. (2004) 29:91–9. 10.1247/csf.29.91 15665504

[66] Lindberg K Amin R Moe OW Hu MC Erben RG Ostman Wernerson A. The kidney is the principal organ mediating klotho effects. J Am Soc Nephrol. (2014) 25:2169–75. 10.1681/ASN.2013111209 24854271 PMC4178446

[67] Forster RE Jurutka PW Hsieh JC Haussler CA Lowmiller CL Kaneko I. Vitamin D receptor controls expression of the anti-aging klotho gene in mouse and human renal cells. Biochem Biophys Res Commun. (2011) 414:557–62. 10.1016/j.bbrc.2011.09.117 21982773 PMC3209523

[68] Melamed ML Michos ED Post W Astor B. 25-hydroxyvitamin D levels and the risk of mortality in the general population. Arch Intern Med. (2008) 168:1629–37. 10.1001/archinte.168.15.1629 18695076 PMC2677029

[69] Friedman DJ Afkarian M Tamez H Bhan I Isakova T Wolf M. Klotho variants and chronic hemodialysis mortality. J Bone Miner Res. (2009) 24:1847–55. 10.1359/jbmr.090516 19419323 PMC2765930

[70] Kurosu H Kuro-o M. The Klotho gene family and the endocrine fibroblast growth factors. Curr Opin Nephrol Hypertens. (2008) 17:368–72. 10.1097/MNH.0b013e3282ffd994 18660672

[71] Imura A Iwano A Tohyama O Tsuji Y Nozaki K Hashimoto N. Secreted Klotho protein in sera and CSF: implication for post-translational cleavage in release of Klotho protein from cell membrane. FEBS Lett. (2004) 565:143–7. 10.1016/j.febslet.2004.03.090 15135068

[72] Hu MC Shi M Zhang J Pastor J Nakatani T Lanske B. Klotho: a novel phosphaturic substance acting as an autocrine enzyme in the renal proximal tubule. FASEB J. (2010) 24:3438–50. 10.1096/fj.10-154765 20466874 PMC2923354

[73] Kurosu H Yamamoto M Clark JD Pastor JV Nandi A Gurnani P. Suppression of aging in mice by the hormone Klotho. Science. (2005) 309:1829–33. 10.1126/science.1112766 16123266 PMC2536606

[74] Hu MC Kuro-o M Moe OW. Renal and extrarenal actions of Klotho. Semin Nephrol. (2013) 33:118–29. 10.1016/j.semnephrol.2012.12.013 23465499 PMC3593734

[75] Razzaque MS Lanske B. The emerging role of the fibroblast growth factor-23-klotho axis in renal regulation of phosphate homeostasis. J Endocrinol. (2007) 194:1–10. 10.1677/JOE-07-0095 17592015 PMC2900827

[76] Craver L Dusso A Martinez-Alonso M Sarro F Valdivielso JM Fernandez E. A low fractional excretion of Phosphate/Fgf23 ratio is associated with severe abdominal Aortic calcification in stage 3 and 4 kidney disease patients. BMC Nephrol. (2013) 14:221. 10.1186/1471-2369-14-221 24119158 PMC3852798

[77] Bachmann S Schlichting U Geist B Mutig K Petsch T Bacic D. Kidney-specific inactivation of the megalin gene impairs trafficking of renal inorganic sodium phosphate cotransporter (NaPi-IIa). J Am Soc Nephrol. (2004) 15:892–900. 10.1097/01.ASN.0000120389.09938.21 15034091

[78] Fernandez AF Sebti S Wei Y Zou Z Shi M McMillan KL. Disruption of the beclin 1-BCL2 autophagy regulatory complex promotes longevity in mice. Nature. (2018) 558:136–40. 10.1038/s41586-018-0162-7 29849149 PMC5992097

[79] Hu MC Shi M Cho HJ Adams-Huet B Paek J Hill K. Klotho and phosphate are modulators of pathologic uremic cardiac remodeling. J Am Soc Nephrol. (2015) 26:1290–302. 10.1681/ASN.2014050465 25326585 PMC4446876

[80] Hu MC Shi M Zhang J Quinones H Griffith C Kuro-o M. Klotho deficiency causes vascular calcification in chronic kidney disease. J Am Soc Nephrol. (2011) 22:124–36. 10.1681/ASN.2009121311 21115613 PMC3014041

[81] Hu MC Shi M Zhang J Quinones H Kuro-o M Moe OW. Klotho deficiency is an early biomarker of renal ischemia-reperfusion injury and its replacement is protective. Kidney Int. (2010) 78:1240–51. 10.1038/ki.2010.328 20861825 PMC3237296

[82] Shi M Flores B Gillings N Bian A Cho HJ Yan S. alphaKlotho mitigates progression of AKI to CKD through activation of autophagy. J Am Soc Nephrol. (2016) 27:2331–45. 10.1681/ASN.2015060613 26701976 PMC4978045

[83] Cannell JJ Grant WB Holick MF. Vitamin D and inflammation. Dermatoendocrinology. (2014) 6:e983401. 10.4161/19381980.2014.983401 26413186 PMC4580066

[84] Mitani H Ishizaka N Aizawa T Ohno M Usui S Suzuki T. *In vivo* klotho gene transfer ameliorates angiotensin II-induced renal damage. Hypertension. (2002) 39:838–43. 10.1161/01.HYP.0000013734.33441.EA 11967236

[85] Izquierdo MC Perez-Gomez MV Sanchez-Nino MD Sanz AB Ruiz-Andres O Poveda J. Klotho, phosphate and inflammation/ageing in chronic kidney disease. Nephrol Dial Transplant. (2012) 4:iv6–10. 10.1093/ndt/gfs426 23258814

[86] Hu MC Shi M Zhang J Addo T Cho HJ Barker SL. Renal production, uptake, and handling of circulating alphaKlotho. J Am Soc Nephrol. (2016) 27:79–90. 10.1681/ASN.2014101030 25977312 PMC4696570

[87] Li YC Kong J Wei M Chen ZF Liu SQ Cao LP. 1,25-Dihydroxyvitamin D(3) is a negative endocrine regulator of the renin-angiotensin system. J Clin Invest. (2002) 110:229–38. 10.1172/JCI0215219 12122115 PMC151055

[88] Vimaleswaran KS Cavadino A Berry DJ Jorde R Dieffenbach AK. Association of vitamin D status with arterial blood pressure and hypertension risk: a mendelian randomisation study. Lancet Diabetes Endocrinol. (2014) 2:719–29. 10.1016/S2213-8587(14)70113-5 24974252 PMC4582411

[89] Rejnmark L Bislev LS Cashman KD Eiriksdottir G Gaksch M Grubler M. Non-skeletal health effects of vitamin D supplementation: a systematic review on findings from meta-analyses summarizing trial data. PLoS ONE. (2017) 12:e0180512. 10.1371/journal.pone.0180512 28686645 PMC5501555

[90] Zhang Z Sun L Wang Y Ning G Minto AW Kong J. Renoprotective role of the vitamin D receptor in diabetic nephropathy. Kidney Int. (2008) 73:163–71. 10.1038/sj.ki.5002572 17928826

[91] Ronco C Cozzolino M. Mineral metabolism abnormalities and vitamin D receptor activation in cardiorenal syndromes. Heart Fail Rev. (2012) 17:211–20. 10.1007/s10741-011-9232-8 21327712

[92] Melenhorst WB Visser L Timmer A van den Heuvel MC Stegeman CA van Goor H. ADAM17 upregulation in human renal disease: a role in modulating TGF-{alpha} availability? Am J Physiol Renal Physiol. (2009) 297:F781–90. 10.1152/ajprenal.90610.2008 19535569

[93] Charbonneau M Harper K Grondin F Pelmus M McDonald PP Dubois CM. Hypoxia-inducible factor mediates hypoxic and tumor necrosis factor alpha-induced increases in tumor necrosis factor-alpha converting enzyme/ADAM17 expression by synovial cells. J Biol Chem. (2007) 282:33714–24. 10.1074/jbc.M704041200 17884817

[94] Mizobuchi M Morrissey J Finch JL Martin DR Liapis H Akizawa T. Combination therapy with an Angiotensin-converting enzyme inhibitor and a vitamin d analog suppresses the progression of renal insufficiency in uremic rats. J Am Soc Nephrol. (2007) 18:1796–806. 10.1681/ASN.2006091028 17513326

[95] Santos RA Simoes e Silva AC Maric C Silva DM Machado RP de Buhr I. Angiotensin-(1-7) is an endogenous ligand for the G protein-coupled receptor Mas. Proc Natl Acad Sci USA. (2003) 100:8258–63. 10.1073/pnas.1432869100 12829792 PMC166216

[96] Vickers C Hales P Kaushik V Dick L Gavin J Tang J. Hydrolysis of biological peptides by human angiotensin-converting enzyme-related carboxypeptidase. J Biol Chem. (2002) 277:14838–43. 10.1074/jbc.M200581200 11815627

[97] Hamming I Cooper ME Haagmans BL Hooper NM Korstanje R Osterhaus AD. The emerging role of ACE2 in physiology and disease. J Pathol. (2007) 212:1–11. 10.1002/path.2162 17464936 PMC7167724

[98] Cui C Xu P Li G Qiao Y Han W Geng C. Vitamin D receptor activation regulates microglia polarization and oxidative stress in spontaneously hypertensive rats and angiotensin II-exposed microglial cells: role of renin-angiotensin system. Redox Biol. (2019) 26:101295. 10.1016/j.redox.2019.101295 31421410 PMC6831892

[99] Sodhi CP Wohlford-Lenane C Yamaguchi Y Prindle T Fulton WB Wang S. Attenuation of pulmonary ACE2 activity impairs inactivation of des-Arg(9) bradykinin/BKB1R axis and facilitates LPS-induced neutrophil infiltration. Am J Physiol Lung Cell Mol Physiol. (2018) 314:L17–31. 10.1152/ajplung.00498.2016 28935640 PMC5866432

[100] Anguiano L Riera M Pascual J Valdivielso JM Barrios C Betriu A. Circulating angiotensin-converting enzyme 2 activity in patients with chronic kidney disease without previous history of cardiovascular disease. Nephrol Dial Transplant. (2015) 30:1176–85. 10.1093/ndt/gfv025 25813276 PMC7107869

[101] Riera M Anguiano L Clotet S Roca-Ho H Rebull M Pascual J. Paricalcitol modulates ACE2 shedding and renal ADAM17 in NOD mice beyond proteinuria. Am J Physiol Renal Physiol. (2016) 310:F534–46. 10.1152/ajprenal.00082.2015 26697977

[102] Bouillon R Carmeliet G Verlinden L van Etten E Verstuyf A Luderer HF. Vitamin D and human health: lessons from vitamin D receptor null mice. Endocr Rev. (2008) 29:726–76. 10.1210/er.2008-0004 18694980 PMC2583388

[103] Wang D Qin H Du W Shen YW Lee WH Riggs AD. Inhibition of S-phase kinase-associated protein 2 (Skp2) reprograms and converts diabetogenic T cells to Foxp3+ regulatory T cells. Proc Natl Acad Sci USA. (2012) 109:9493–8. 10.1073/pnas.1207293109 22645357 PMC3386130

[104] Gassen NC Niemeyer D Muth D Corman VM Martinelli S Gassen A. SKP2 attenuates autophagy through Beclin1-ubiquitination and its inhibition reduces MERS-Coronavirus infection. Nat Commun. (2019) 10:5770. 10.1038/s41467-019-13659-4 31852899 PMC6920372

[105] Morange PE Tregouet DA Godefroy T Saut N Bickel C Rupprecht HJ. Polymorphisms of the tumor necrosis factor-alpha (TNF) and the TNF-alpha converting enzyme (TACE/ADAM17) genes in relation to cardiovascular mortality: the AtheroGene study. J Mol Med. (2008) 86:1153–61. 10.1007/s00109-008-0375-6 18600307

[106] Horiuchi K Kimura T Miyamoto T Takaishi H Okada Y Toyama Y. Cutting edge: TNF-alpha-converting enzyme (TACE/ADAM17) inactivation in mouse myeloid cells prevents lethality from endotoxin shock. J Immunol. (2007) 179:2686–9. 10.4049/jimmunol.179.5.2686 17709479

[107] Dusso A Arcidiacono MV Yang J Tokumoto M. Vitamin D inhibition of TACE and prevention of renal osteodystrophy and cardiovascular mortality. J Steroid Biochem Mol Biol. (2010) 121:193–8. 10.1016/j.jsbmb.2010.03.064 20359533 PMC2906659

[108] Gallieni M Kamimura S Ahmed A Bravo E Delmez J Slatopolsky E. Kinetics of monocyte 1 alpha-hydroxylase in renal failure. Am J Physiol. (1995) 268:F746–53. 10.1152/ajprenal.1995.268.4.F746 7733332

[109] Kapustin AN Chatrou ML Drozdov I Zheng Y Davidson SM Soong D. Vascular smooth muscle cell calcification is mediated by regulated exosome secretion. Circ Res. (2015) 116:1312–23. 10.1161/CIRCRESAHA.116.305012 25711438

[110] Cordes KR Sheehy NT White MP Berry EC Morton SU Muth AN. miR-145 and miR-143 regulate smooth muscle cell fate and plasticity. Nature. (2009) 460:705–10. 10.1038/nature08195 19578358 PMC2769203

[111] Cheng Y Liu X Yang J Lin Y Xu DZ Lu Q. MicroRNA-145, a novel smooth muscle cell phenotypic marker and modulator, controls vascular neointimal lesion formation. Circ Res. (2009) 105:158–66. 10.1161/CIRCRESAHA.109.197517 19542014 PMC2728297

[112] Doberstein K Steinmeyer N Hartmetz AK Eberhardt W Mittelbronn M Harter PN. MicroRNA-145 targets the metalloprotease ADAM17 and is suppressed in renal cell carcinoma patients. Neoplasia. (2013) 15:218–30. 10.1593/neo.121222 23441135 PMC3579323

[113] De Ciuceis C Amiri F Brassard P Endemann DH Touyz RM Schiffrin EL. Reduced vascular remodeling, endothelial dysfunction, and oxidative stress in resistance arteries of angiotensin II-infused macrophage colony-stimulating factor-deficient mice: evidence for a role in inflammation in angiotensin-induced vascular injury. Arterioscler Thromb Vasc Biol. (2005) 25:2106–13. 10.1161/01.ATV.0000181743.28028.57 16100037

[114] Norlander AE Madhur MS Harrison DG. Correction: the immunology of hypertension. J Exp Med. (2018) 215:719. 10.1084/jem.2017177301022018c 29305396 PMC5789420

[115] Oh J Matkovich SJ Riek AE Bindom SM Shao JS Head RD. Macrophage secretion of miR-106b-5p causes renin-dependent hypertension. Nat Commun. (2020) 11:4798. 10.1038/s41467-020-18538-x 32968066 PMC7511948

